# Filtering of MS/MS data for peptide identification

**DOI:** 10.1186/1471-2164-14-S7-S2

**Published:** 2013-11-05

**Authors:** Jason Gallia, Katelyn Lavrich, Anna Tan-Wilson, Patrick H Madden

**Affiliations:** 1 SUNY Binghamton Computer Science Department, Binghamton, NY, USA; 2 SUNY Binghamton Biological Sciences Department, Binghamton, NY, USA

## Abstract

**Background:**

The identification of proteins based on analysis of tandem mass spectrometry (MS/MS) data is a valuable tool that is not fully realized because of the difficulty in carrying out automated analysis of large numbers of spectra. MS/MS spectra consist of peaks that represent each peptide fragment, usually *b *and *y *ions, with experimentally determined mass to charge ratios. Whether the strategy employed is database matching or *De Novo *sequencing, a major obstacle is distinguishing signal from noise. Improved ability to distinguish signal peaks of low intensity from background noise increases the likelihood of correctly identifying the peptide, as valuable information is preserved while extraneous information is not left to mislead.

**Results:**

This paper introduces an automated noise filtering method based on the construction of orthogonal polynomials. By subdividing the spectrum into a variable number (3 to 11) of bins, peaks that are considered "noise" are identified at a local level. Using a *De Novo *sequencing algorithm that we are developing, this filtering method was applied to a published dataset of more than 3000 mass spectra and an original dataset of more than 300 spectra. The samples were peptides from purified known proteins; therefore, the solutions could be compared to the correct sequences and the peaks corresponding to *b*, *y *and other fragments of significance could be identified. The same procedure was applied using two other published filtering methods. The ratios of the number of significant peaks that were preserved relative to the total number of peaks in each spectrum were determined. In the event that filtering out too many or too few signal peaks can lead to inaccuracy in sequence determination, the percentage of amino acid residues in the correct positions relative to the total number of amino acid residues in the correct sequence was also calculated for each sequence determined.

**Conclusions:**

The results show that an orthogonal polynomial-based method of distinguishing signal peaks from background in mass spectra preserves a greater portion of signal peaks than compared methods, improving accuracy in sequence determination.

## Background

**Figure 1:**
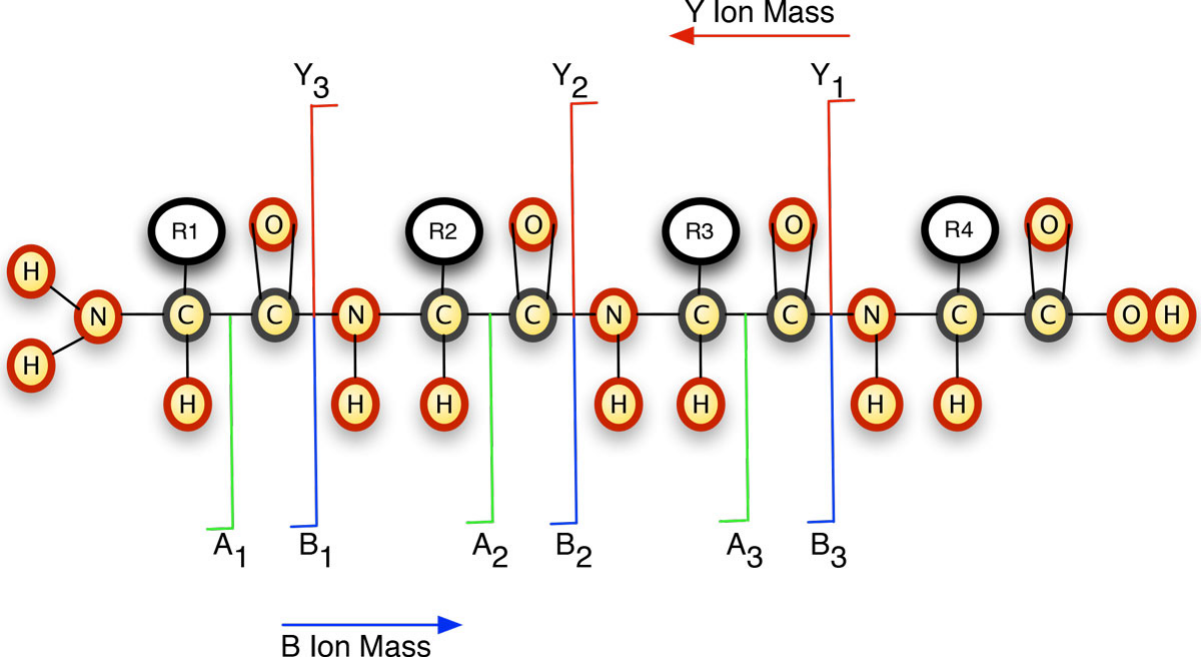
**Typical peptide fragmentation generates *b *or *y *ions of different mass to charge ratios, and also *a *ions**. Correspondence of the experimentally determined masses to the molecular masses of the amino acid residues can be used to derive the sequence of the parent ion.

Proteins carry out the functions of cells; so information on the proteome, i.e., the complement of proteins and the changing levels of individual proteins, can serve as the basis for understanding natural and disease-related cellular processes. Tandem mass spectrometry (MS/MS) has become a staple approach for defining the proteome. After cleaving proteins to be identified into peptides, the resulting sets of peptides can be analyzed quickly and relatively inexpensively in "shotgun" fashion; large numbers of peptides are separated by their mass to charge ratio (m/z) and fragmented sequentially, producing mass spectra that can be analyzed. The identity of the protein is inferred from the identification of the peptide which in turn is based on the experimental determination of the masses of the whole peptide and its fragments in the mass spectrometer. One at a time, peptide ions having the same m/z are sequestered, fragmented, and the m/z of the fragment ions determined. Ideally, the fragmentation occurs randomly at one of the peptide bonds, generating a series of *b *and *y *ions (Figure 1).

**Figure 2:**
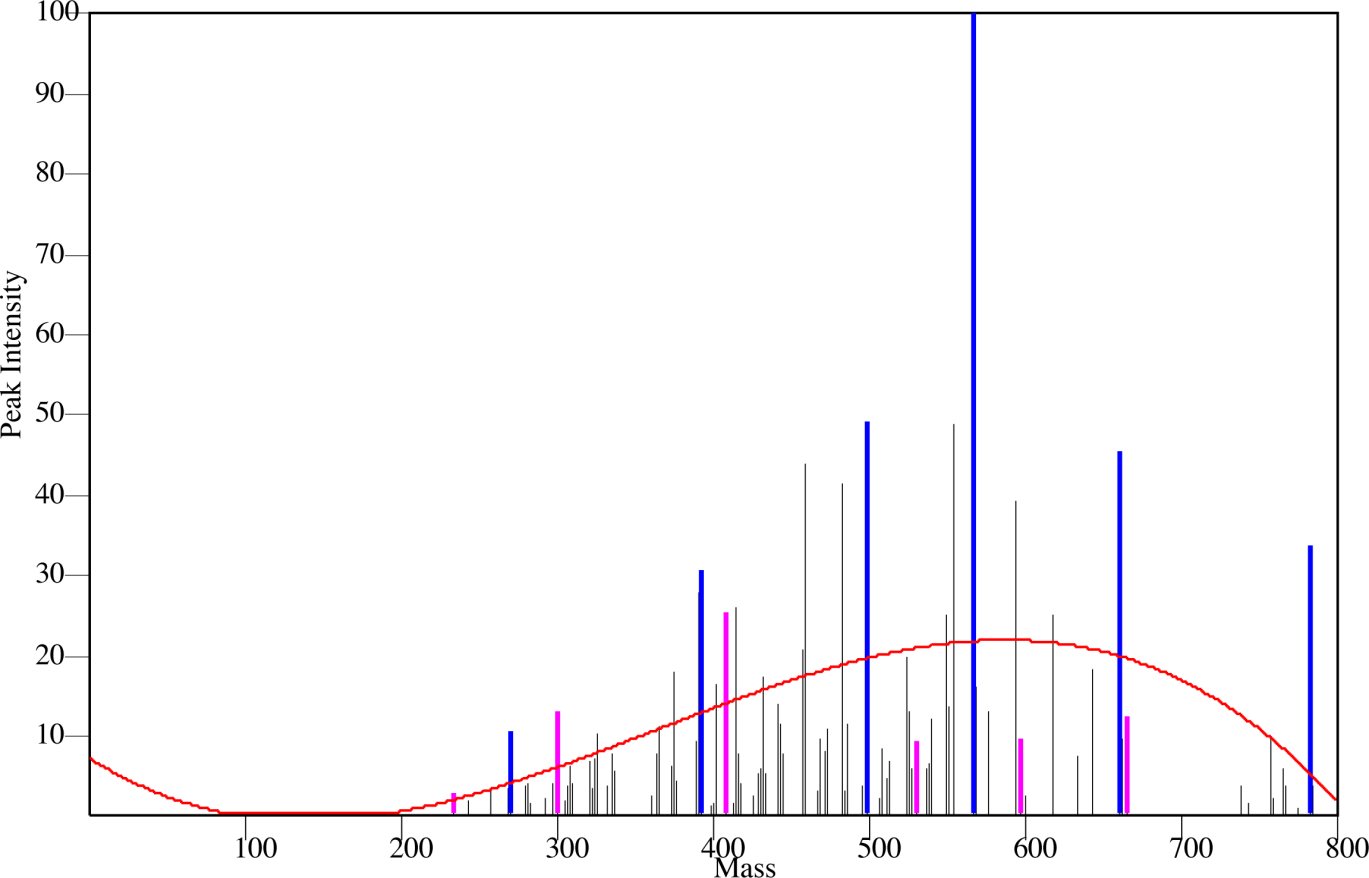
**An MS/MS data set that has been filtered through the use of eleven bins and an orthogonal polynomial with a degree of three**.

Working backwards from the mass spectra to decipher the nature of the parent peptide, and by inference the protein, can be challenging, and this has been the focus of many research groups. One common approach is database matching: the spectra obtained experimentally are compared against virtual mass lists generated from known protein sequences. Gaining in popularity is *De Novo *sequencing, where algorithmic methods are used to determine the amino acid sequence of the parent peptide from the spacing and positions of spectra peaks, which correspond to the m/z of the peptide fragments. When examining spectra, it is common to look for the peaks with intensities that are much higher compared to the background (Figure 2) as such peaks are more likely to correspond to the *b *and *y *ions[1]. This correlation does not always hold, however, so that simply eliminating peaks based on a ranking by intensity may cull data that is crucial for identification. In a characteristic collisional-induced dissociation (CID) spectrum, the fifth most abundant fragment has intensity ten times lower than that of the most abundant fragment[1].

Fragmentation can also occur at other points on the peptide. A common fragment generated by collisional-induced dissociation, for instance, is the *a *ion, which has a mass lower by 28 amu compared to the nearest *b *ion as it has also lost the C = O. Fragments that have amino acid residues with a hydroxyl group may lose H_2_O (-18 amu), those that have amino groups may lose NH_3 _(-17 amu). The recognition of these additional fragments can help to distinguish one sequence over another. When peaks corresponding to these fragments are of low intensity, however, it can be difficult to distinguish between valuable information and background noise.

No matter what method of peptide identification is used, there is room for improvement. When four database search methods were compared, there was good agreement on half of the proteins identified in total [2]. In another report, 69,978 tandem mass spectra were obtained for a mixture of known proteins. Of these, only 5,678 were able to be identified by the database matching program SEQUEST[1]. In a review of 13 de novo sequencing methods, Allmer[3] points out that with current instrumentation and algorithms, an all-or-nothing score, ie. correct or wrong sequence, may be too harsh because very few sequences would be 100% correct. Accuracy of identification is low because noise in the experimental data is a significant challenge. This is exacerbated when fragment ions that should theoretically exist are not detected or are of such low intensity that they cannot be distinguished from noise. Choo and Tham[4] illustrate the impact of background noise when they calculated a quality score (QS) from self-convolution of the mass spectra for a theoretical 14-residue peptide subjected to different degradation processes. They showed that removal of 8 *y *ion peaks (with no Gaussian noise) reduced the QS by only 43% whereas increasing white Gaussian noise from 0 to 30% (keeping the full complement of *b *and *y *ion peaks) resulted in an 80% drop in their calculated QS. Clearly, background noise poses a significant challenge to recognition especially of low-intensity peaks.

When thousands of MS/MS spectra are generated in a single experiment, removal of poor quality spectra from analysis can be time-saving. This was the approach taken by Flikka et al.[5] who used machine learning to distinguish between good and bad spectra by using a number of spectral features. Many such methods for selecting spectra to analyze such as the one used by Wong et al[6] are partially based on the signal to noise ratio. Simple methods for eliminating noise, such as removing all peaks below a certain threshold or selecting only a fixed number of the highest peaks have been explored. Purvine et al[7] proposed a method to distinguish signal from noise. They sorted intensity values of the peaks in descending order, and defined the median value of the lower half of the intensity values to be the noise level. Also with the goal of recognizing poor quality spectra, Xu and Freitas[8] first sort peaks according to intensity, from lowest to highest. The lowest intensity is considered to be noise. The algorithm starts by calculating the Signal to Noise Ratio (SNR) for the second peak, comparing that to the SNR threshold set by the user. If the SNR value for a given peak is higher than that set by the user, the noise level is set to that peak. Using database searching techniques, this algorithm was demonstrated to work better than other filtering approaches. A large percentage (76-91%) of MS/MS spectra that did not yield a peptide match were removed, while only 3.6-9.4% of those that yielded a peptide match were removed. In these examples, the intensity of background noise is determined and applied uniformly across the spectrum. As noise levels vary across a spectrum, these methods can be too aggressive in some areas, while not being aggressive enough in others.

Ding et al[9] takes into consideration changes in SNR in different regions of the spectrum. Intensities of peaks whose m/z values indicate relationships such as complementary *b *and *y *ions, fragments with and without loss of water or ammonia are adjusted upward, after which peaks that represent maxima in local regions of the spectrum are extracted. All other peaks that are presumed to be noise are eliminated. The de-noising method removes about 69% of peaks in a spectrum, and increased the number of spectra that could be identified by 14-31%, depending upon the dataset used. Using such a linear combination approach and applying different weights to the intensity adjustment of the peaks, Lin et al[10] were able to increase the success of identification by a further 14-23%. We propose an alternative approach that allows for noise levels to be automatically adjusted across an entire spectrum in a continuous manner.

The focus of this paper is on a new method to filter noise across an entire spectrum by modeling peak intensity using orthogonal polynomials. We designate different regions of each spectrum, giving the user a choice of how many such regions (bins) to set. We filter out noise through construction of orthogonal polynomials to fit a curve to the noise level in separate bins. We demonstrate the effectiveness of the approach using spectra generated from peptides of proteins with known sequences. We track the number of significant peaks (which correspond to primary fragment ions) while still noting but preserving the non-signficant peaks during filtering. This new filtering method is compared to the filtering methods of Purvine et al.[7] and of Xu and Freitas[8] by application to the large dataset generated from a mixture of purified proteins[11] and an original dataset for one protein that was generated in-house. Using a de novo sequencing method that we are developing, we assess the impact of our filtering method with the published methods on the accuracy of the amino acid sequences ascribed to each peptide.

## A new filtering method

If a mass spectrum were to contain only a full complement of *b *or *y *ions, peptide identification would be trivial; one could simply calculate the difference between peaks. In practice, spectra are cluttered with an abundance of peaks representing a wide range of possible fragmentations or modifications.

One might note, however that the minimum mass difference between a pair of *b *ions, or a pair of *y *ions, is equal to the mass of the smallest amino acid - and in general, the difference will be larger than this minimum. The sequences of *b *and *y *ions are overlaid, but one can be assured that these primary ions will not be clustered together, and that typically, they will dominate their local neighborhoods.

Noise levels can vary across a spectra; to separate significant ions from noise, the degree of filtering should also vary. The method pursued by our group can be seen as an extension of a recent method for filtering of data proposed by Xu[8]. The peaks of the spectrum are sorted from smallest to largest based on intensity, ${\hat{I}}_{k}$ where *k *= 1, 2, 3*, . . . , N*. These peaks are referred to as *A_n_*, and we use the naming scheme used by Xu.

The Xu algorithm finds noise levels by using the peaks in the data set combined with linear regression to calculate the SNR. This process starts by examining the second smallest peak and calculating the SNR ratio. Because there is only one peak available, a user defined value referred to as *ρ *is used to calculate the SNR.

$${\hat{I}}_{2}=(1+\rho )*I_{1}$$

$$SNR=\frac{I_{k}}{{\hat{I}}_{k}}$$

If the SNR ratio is greater than a user defined *SNR_min_*, then the process is halted, and the noise level for that bin is set at *I*_1_. However, if the SNR ratio is not greater than *SNR_min_*, peaks 3 to *N *are examined using a linear regression model described by Xu[8]. Each peak is examined in order until the calculated *SNR *is greater then the *SNR_min_*. The equations for this are as follows:

$$\begin{array}{lll} \;\;{\hat{I}}_{k} & = & \alpha *k+\beta \\ \left[\begin{array}{c} \hat{\alpha }\\ \hat{\beta } \end{array}\right] & = & {\left(A^{T}A\right)}^{-1}A^{T}I \end{array}\;\;$$

$$A=\left[\begin{array}{cc} 1 & 1\\ 2 & 1\\ \vdots & \vdots \\ k-1 & 1 \end{array}\right]\;\text{and}\;I=\left[\begin{array}{c} I_{1}\\ I_{2}\\ \vdots \\ I_{k-1} \end{array}\right]$$

where *α *and *β *are linear regression parameters.

### Orthogonal polynomials

The algorithm by Xu proved to be effective for many of the spectra we examined, but for others, it was easy to identify cases where the noise levels were either too high or too low. In practice, the level of noise differs across spectra, and also the masses at which noise levels change with a spectrum vary. This observation indicated that using a single noise level over a spectra was not ideal; we first adapted the Xu approach to use a set of equally spaced bins, with each bin being assigned an individual noise level.

With multiple distinct noise levels, we saw a number of problematic situations. There were discontinuities between adjacent bins, where noise levels could vary considerably. To address these shortcomings, we pursued an approach for fitting a curve to the calculated noise levels; this allowed for smooth changes along the x-axis.

One well known numerical technique to fit a curve to a set of data points is to use a *k^th ^*order orthogonal polynomial. In order to do this, a model for our problem was defined as

$$Y=\overset{k}{\underset{j=0}{\sum }}\beta_{j}\psi_{j}\left(X_{j}\right)$$

with k being the polynomial degree. The least squares estimate of parameters, under an orthogonal polynomial, defined as:

$${\hat{\beta }}_{j}=\frac{\sum_{i=0}^{n}Y_{i}\psi_{j}(X_{i})}{\sum_{i=0}^{n}\psi_{j}^{2}(X_{i})}$$

Because our data is equally spaced, and the mass values can be transformed by the equation

$$\begin{array}{c} \text{half - range }t=1\left(1\right)\left[\frac{n+1}{2}\right]\\ \;\;\;\;\;\;\;\;\;\;\;\;\;\;\;\;\;\;\;\;\;\;\;\;\;\;\;\;\;\;\;\;\;\;\;\;\;\;\;\;\;\;\;\;\;\;\;\;\;\;\;\;\;\;x_{i}=i=\frac{x_{i}-x_{t}}{\text{difference between points}} \end{array}$$

the system of orthogonal polynomials ascribed to Chebyshev can be used [12,13]:

$$\begin{array}{l} \psi_{0}=1\\ \psi_{1}=\lambda_{1}x\\ \psi_{2}=\lambda_{2}(x^{2}-\frac{1}{12}(n^{2}-1))\\ \psi_{3}=\lambda_{3}(x^{3}-\frac{1}{20}(3n^{2}-1)x)\\ \psi_{4}=\lambda_{4}(x^{4}-\frac{1}{14}(3n^{2}-13)x+\frac{3}{560}(n^{2}-1)(n^{2}-9)) \end{array}$$

The values for *λ_r _*are chosen such that the tabulated values for all *ψ_j_*'s are positive or negative integers, while *n *is the number of data points used. These polynomials have been extensively calculated by Pearson and Hartley with their calculations being used in our experiments[14]. The method, and notation, are typical for this type of numerical analysis.

Once ${\hat{\beta }}_{j}$'s have been tabulated, the filtering level at each of the recorded masses is then calculated. This is done by first transforming each of the masses defined by equation 1 above. These transformed masses are then placed into the orthogonal polynomial equation with the result being the noise level at that mass. An example of a noise level created in this manner can be seen in Figure 2.

## Experimental results

In this section, we demonstrate the impact of our new filtering approach. We have performed a series of experiments using spectrum data obtained from our research lab (using known samples, or those identified with a high degree of confidence), and also samples obtained from the PEAKS research group[15], the Pepnovo research group[16], and two data sets provided by Keller et al[11].

Our first set of experiments tracks the percentage of primary peaks and secondary peaks compared to noisy peaks. Primary peaks are associated with any *b *or *y *ion, while secondary peaks are any *a *ions, or primary peaks coupled with a loss of water or loss of ammonia. Noisy peaks are any peaks that are not classified as either primary or secondary. Some data sets have been centroided (tight clusters of peaks are grouped together). Both centroided and non-controided data sets were used to illustrate that our method can be used to filter a variety of different data. For data sets that were not centroided, all peaks within 0.1 amu were removed from the number of noisy peaks since they could be considered to be a primary or secondary peaks themselves. We use this metric, as it is similar to the work performed by Bern[1].

In a second set of experiments, we use our *De Novo *sequencing approach[17], with the spectra pre-processed by the variety of filtering methods. The results are reported with tables that show the number of identified amino acids over the whole data set.

### Data sets considered

To evaluate the different filtering methods, experiments were first run against a total of 3105 spectra from the previously mentioned sources and 384 spectra generated in-house. Each set of spectra contained a different amount of initial noise as well as primary and secondary ions. Table 1 illustrates the initial nature of the data sets. For the data sets generated in-house, the primary and secondary peaks constitute 1.23% of all observed peaks. By eliminating the lowest 10% from consideration, the percentage rises to 4.77%. If one keeps only the highest 10% of peaks, the percentage of primary peaks rises to 6.97%. It should also be noted that the Keller and Pepnovo data have been centroided. This has significantly decreased the number of peaks in each of their data sets, resulting in higher percentages Table 1.

**Table 1:** Percent of primary and secondary peaks compared to noise if a percentage of the highest intensity peaks are kept

Data Set					
**Percent Kept**	**Binghamton**	**Omics A**	**Omics B**	**Peaks Data**	**Pepnovo Data**

Top 100%	1.23	16.34	17.37	0.73	17.64
Top 90%	4.77	17.58	18.65	2.71	18.92
Top 70%	4.77	20.00	21.45	2.71	22.32
Top 50%	4.77	23.19	25.69	2.71	27.76
Top 30%	4.90	31.45	33.40	3.07	38.35
Top 10%	6.97	49.00	50.87	5.88	66.48

There are a few important points to highlight with regards to the data. First, for any m/z value, there can only be an integer number of ions observed; a spectrum is in essence a discrete histogram. As one adjusts the "noise threshold," there can be large jumps in the number of eliminated peaks. Second, as the filtering becomes more aggressive, and peaks are eliminated, the percentage of primary and secondary peaks increases - but it is frequently the case some of these valuable peaks are lost. To carry this to an extreme: if one were to filter, and retain only a single *y *ion - 100% of the remaining peaks would be primary in nature, but peptide identification would be nearly impossible.

### Comparison of filtering methods

For comparison, we first evaluated a filtering technique developed by Purvine *et al*[7], known as Spequal. This uses a single set of filtering parameters. The Spequal method eliminated 14.85% of the primary ion peaks and 70.94% of the noisy peaks; it does not support SNR adjustment.

Both the Xu algorithm, and the approach presented in this paper were applied with SNR ratios of 1, 3, 5, 7, 9 and 11. This range of SNR ratios was chosen, as they vary from being very gentle to very aggressive, giving a good overview of the trends.

For our approach, bin sizes of 3, 5, 7, 9 and 11 were used. The use of three bins results in broad, smooth curves. As most peptides encountered had between five and twelve amino acids, there is little to be gained by having a higher number of bins - the curve fitting approach works best if there are primary ions in each bin. To adjust the flexibility of orthogonal polynomial curve fitting, polynomials with degree 2, 4, 5, 5 and 5 were used for each of the respective bin sizes.

There are constraints on the polynomial degrees - for a case with only three bins, one can have at most a degree two polynomial. As the number of bins increases, more complex polynomials become possible; at most, the degree can be one less than the number of bins. As the number of bins increases, however, we observed diminishing impact of higher degree polynomials - beyond degree 5, we saw no significant change in the shape of the curves.

The results of these experiments can be found in Tables 2, 3, 4, 5 and 6. In comparing our approach against the Xu and Purvine methods, our approach was able to produce a better peak to noise percentage in every data set except for the Peaks data set. For the Peaks data set, the Xu method produced the best results. On average, our method was able to retain twice as many primary ions than the Xu method.

**Table 2:** Percentage of primary and secondary peaks compared to noise from different filtering methods on data generated by our Biology Department

Method Applied							
***SNR_min_***	**Purvine**	**Xu**	**Ortho Poly** **3 Bins**	**Ortho Poly** **5 Bins**	**Ortho Poly** **7 Bins**	**Ortho Poly** **9 Bins**	**Ortho Poly** **11 Bins**

1	4.74	4.74	4.49	4.78	4.35	1.64	3.82
3	4.74	9.11	10.15	8.42	5.69	3.84	6.97
5	4.74	9.46	11.66	9.34	5.48	3.88	7.19
7	4.74	7.62	12.10	9.20	5.82	3.96	7.70
9	4.74	7.35	12.53	9.17	6.27	3.74	7.55
11	4.74	6.66	12.58	8.79	6.42	3.79	7.28

**Table 3:** Percentage of primary and secondary peaks compared to noise from different filtering methods on data from the Keller A mixture

Method Applied							
***SNR_min_***	**Purvine**	**Xu**	**Ortho Poly** **3 Bins**	**Ortho Poly** **5 Bins**	**Ortho Poly** **7 Bins**	**Ortho Poly** **9 Bins**	**Ortho Poly** **11 Bins**

1	15.43	14.32	13.99	14.53	14.58	15.35	15.35
3	15.43	14.47	15.36	16.49	19.77	23.21	23.21
5	15.43	14.28	15.27	16.39	20.80	24.60	24.60
7	15.43	14.19	15.17	16.18	20.70	24.79	24.79
9	15.43	14.15	15.17	16.11	20.90	25.39	25.39
11	15.43	14.13	15.16	16.08	21.05	25.57	25.57

**Table 4:** Percentage of primary and secondary peaks compared to noise from different filtering methods on data from the Keller B mixture

Method Applied							
***SNR_min_***	**Purvine**	**Xu**	**Ortho Poly** **3 Bins**	**Ortho Poly** **5 Bins**	**Ortho Poly** **7 Bins**	**Ortho Poly** **9Bins**	**Ortho Poly** **11 Bins**

1	16.54	14.77	15.22	15.52	15.71	16.59	16.59
3	16.54	15.67	18.44	19.59	23.08	26.27	26.27
5	16.54	15.20	18.82	19.89	24.82	28.21	28.21
7	16.54	14.75	18.56	19.26	24.33	28.48	28.48
9	16.54	14.51	18.55	19.13	24.74	29.35	29.35
11	16.54	14.29	18.44	18.91	25.16	29.71	29.71

**Table 5:** Percentage of primary and secondary peaks compared to noise from different filtering methods on data obtained from Peaks group

Method Applied							
***SNR_min_***	**Purvine**	**Xu**	**Ortho Poly** **3 Bins**	**Ortho Poly** **5 Bins**	**Ortho Poly** **7 Bins**	**Ortho Poly** **9 Bins**	**Ortho Poly** **11 Bins**

1	1.82	2.71	2.58	1.96	1.16	1.01	0.87
3	1.82	8.78	7.87	5.93	7.20	7.26	8.64
5	1.82	14.24	9.96	9.60	8.82	11.40	10.86
7	1.82	19.08	10.47	7.61	9.58	12.19	13.71
9	1.82	17.10	10.31	10.04	11.68	15.30	14.74
11	1.82	17.55	10.24	10.83	10.71	15.77	15.29

**Table 6:** Percentage of primary and secondary peaks compared to noise from different filtering methods on data obtained from the Pepnovo group

Method Applied							
***SNR_min_***	**Purvine**	**Xu**	**Ortho Poly** **3 Bins**	**Ortho Poly** **5 Bins**	**Ortho Poly** **7 Bins**	**Ortho Poly** **9 Bins**	**Ortho Poly** **11 Bins**

1	21.13	18.04	18.61	18.91	18.60	19.05	19.69
3	21.13	23.53	26.59	28.08	35.55	42.38	46.62
5	21.13	23.27	27.14	28.29	38.65	45.75	50.75
7	21.13	21.61	26.38	28.56	38.73	46.35	50.56
9	21.13	20.55	26.14	27.62	38.62	45.10	51.34
11	21.13	19.84	25.96	26.29	38.55	45.18	51.37

### Protein and peptide identification

The end goal for mass spectrometry is the identification of peptides and proteins; the filtering of data is a means to that end. In this section, we detail experiments performed by our *De Novo *approach, to show the impact of the filtering methods.

In order to make the nature of our experiments clear, we will be precise on the metrics used to evaluate an identification.

Any sequence compared against a known reference can be evaluated in terms of amino acids that *match *in both sequences, and at the appropriate mass position. To be specific, we give the following example.

Suppose that the original peptide was the sequence "ABC," while the sequence suggested by the *De Novo *algorithm was "WC," with the mass of AB being equal to W. The two sequences match on one amino acid, "C." The proposed sequence would be scored as being 33% correct. An alternative proposed sequence "CW" would not match on any amino acids, as the common *C *is not at the same position in terms of peptide mass. Similarly, a proposed sequece of "CAB" would also have no matching amino acids.

Note that if the correct sequence was "WC", while the proposed sequence was "ABC," the match would be 50% correct, with the correct sequence containing two amino acids. For consistency, we use the number of amino acids in the known sequence as the denominator, while the number of correctly matching amino acids is the numerator.

In all cases, the mass of a sequence suggested by an identification method should match that of the reference sequence (subject to the error tolerance of the equipment).

The mass (and number of amino acids) present in the reference sequence has a significant impact on identification accuracy; the following tables illustrate this. Our *De Novo *sequencing approach produces a *set *of possible identifications; we report the best match from the top 10 scored sequences. In practice, *De Novo *predictions are compared to databases of known peptides - it is generally not necessary to get an exact match, but rather get "close enough" for complete identification. With close matches, a database method could be used to help further the identification process.

The first set of results can be seen in Tables 7 and 8. These tables show the average percent of the amino acid chain identified over the entire data set. In each of these tables, our approach was able to identify, on average, more of the amino acid chain than the previous methods. However, what is interesting to note is which of the configurations produced the best results. For the Peaks data, it came from using a setup of 3 bins with an orthogonal polynomial of degree 2 and a SNR ratio of 7. By contrast, the best results from PepNovo data were obtained using a set up of 11 bins with an orthogonal polynomial of degree 5 and a SNR of 5. These two experiments illustrate the merit of being able to tune a filtering method to data; given the diversity of spectrometry equipment, it is unlikely that there is any single "best" approach.

**Table 7:** Average percent of the amino acid chain identified on the Peaks data set

Method Applied							
***SNR_min_***	**Purvine**	**Xu**	**Ortho Poly** **3 Bins**	**Ortho Poly** **5 Bins**	**Ortho Poly** **7 Bins**	**Ortho Pol** **9 Bins**	**Ortho Poly** **11 Bins**

1	20.56	18.92	19.63	20.24	15.58	10.56	12.69
3	20.56	16.67	16.40	10.83	14.46	13.52	13.72
5	20.56	21.56	15.27	18.05	16.20	17.95	16.59
7	20.56	21.42	24.90	17.50	19.49	16.10	17.69
9	20.56	18.00	14.64	17.19	22.22	15.66	19.13
11	20.56	19.41	17.04	16.99	17.70	16.31	21.02

**Table 8:** Average percent of the amino acid chain identified on the Pepnovo data set

Method Applied							
***SNR_min_***	**Purvine**	**Xu**	**Ortho Poly** **3 Bins**	**Ortho Poly** **5 Bins**	**Ortho Poly** **7 Bins**	**Ortho Pol** **9 Bins**	**Ortho Poly** **11 Bins**

1	19.95	7.51	8.21	21.07	20.12	20.69	20.26
3	19.95	7.84	9.63	23.28	23.87	22.42	24.40
5	19.95	7.57	8.65	22.93	22.62	23.43	24.85
7	19.95	7.76	7.68	20.74	20.76	22.23	23.52
9	19.95	7.69	8.21	21.20	20.63	20.17	23.75
11	19.95	7.71	19.49	20.98	21.79	20.13	24.32

In our final set of experiments, we performed the same sequencing tests on the data provided to us from the Keller group. These results can be found in Tables 9 and 10. Again, it can be seen that the filtering method provided in this paper allowed for more accurate peptide identification. We note, however, that the identification accuracy is fairly low. After examining the spectra and the identifications, it appears that the data sets have a rather high error tolerance for both spectrum peaks as well as precursor mass. Our *De Novo *sequencing method is relatively sensitive to this; we are working on methods to make our approach more robust.

**Table 9:** Average percent of the amino acid chain identified on the Keller data set

Method Applied							
***SNR_min_***	**Purvine**	**Xu**	**Ortho Poly** **3 Bins**	**Ortho Poly** **5 Bins**	**Ortho Poly** **7 Bins**	**Ortho Pol** **9 Bins**	**Ortho Poly** **11 Bins**

1	3.08	2.97	3.41	2.88	2.81	2.63	3.17
3	3.08	2.93	4.35	3.13	3.24	3.38	3.78
5	3.08	2.91	4.19	2.96	3.10	3.18	3.39
7	3.08	2.90	3.48	2.71	3.08	3.20	3.29
9	3.08	3.07	2.66	2.75	2.98	2.95	3.04
11	3.08	4.31	2.58	2.71	2.80	2.80	2.88

**Table 10:** Average percent of the amino acid chain identified on the Keller B data set

Method Applied							
***SNR_min_***	**Purvine**	**Xu**	**Ortho Poly** **3 Bins**	**Ortho Poly** **5 Bins**	**Ortho Poly** **7 Bins**	**Ortho Pol** **9 Bins**	**Ortho Poly** **11 Bins**

1	3.40	3.04	3.48	3.04	2.86	3.02	2.83
3	3.40	2.76	3.47	2.70	3.17	2.96	3.74
5	3.40	2.63	3.42	2.67	2.84	2.79	2.90
7	3.40	2.66	3.15	2.49	2.89	2.73	2.85
9	3.40	2.66	2.93	2.42	2.86	2.77	2.64
11	3.40	2.71	2.89	2.34	2.75	2.91	2.53

Though our method produced the best result in Tables 7 through 10, the difference between our method compared to the other methods was not always significant. This can be easily seen in Table 10 where the difference between the best percentage from our method and the percentage from the Purvine method is 0.08%. Even small improvements are beneficial, however: when using *De Novo *sequencing in combination with a database search tool such as blast, better initial sequencing results lead to more accurate BLAST results[18].

## Summary and conclusion

The work presented in this paper is an expansion upon prior work described in an extended abstract[19]. Additional data sets have been considered, the description of our orthogonal polynomial approach has been expanded, and comparisons are made with a number of other methods.

In this paper, we have focused on filtering methods for MS/MS spectra. Higher peaks are indicative of "primary" ions, but peak levels can vary considerably across a spectrum. Simple fixed value cuts can remove important peaks, while step functions can introduce abrupt discontinuities.

The approach we have pursued here constructs an orthogonal polynomial function, which gently adjusts to track average peak intensities across a spectrum. Using this method, we are able to filter by intensity at a "local" level - this helps preserve peaks at higher mass values, which can be critical for correct identifications.

On experiments with data obtained by our research group, and from the group which developed the PEAKS research group, we find that our method preserves a greater percentage of *b *and *y *ions compared to the prior method by Xu. By adjusting the number of bins, and the desired SNR ratio, the aggressiveness of our approach can be tuned easily.

In experiments with our *De Novo *identification approach, the filtering improves results. Across a wide range of spectra, top scoring sequences produced algorithmically matched the known peptide sequences.

Experiments with a number of different approaches, described in Tables 7 through 10, allow for a few observations. First, we observe that the Purvine method works well in many cases, and is consistent; a shortcoming is relatively little ability to adjust to incoming data. The Xu method is more flexible, and can adjust noise levels, but performance can vary considerably, as evident in Tables 7 and 8. The spectra contained in the PepNovo data set have relatively uniform peak heights, with little variation between noise and signal; this proved challenging for the Xu method. The approach we describe improves on this work, providing effective filtering across a wide range of spectra.

## Methods

### Sample preparation by the research group

Tryptic peptides of bovine serum albumin were resolved on a Microhm C18 AQ column (0.075 × 150 mm) set in-line to a nanospray unit on a QStar-XL Q-TOF mass spectrometer (AB Sciex, Framingham, MA) operating in information-dependent acquisition mode.

## Competing interests

The authors declare that they have no competing interests.

## Authors' contributions

Jason Gallia designed, applied and tested the orthogonal polynomial-based filtering method, wrote the *De Novo *sequencing program, and wrote the Methods and Results sections; Katelyn Lavrich studied the *De Novo *sequencing program and suggested changes that improved the program substantially and analyzed the dataset using the commercial Peaks program; Anna Tan-Wilson defined the *De Novo *sequencing problem and provided ongoing consultation and one of the datasets, guided the work of Katelyn Lavrich, and wrote the Background; Patrick Madden as principal mentor to Jason Gallia guided all aspects of the work and prepared the final draft of the paper. All authors read and approved the final manuscript.

## Declarations

Publication of this article was supported by the State University of New York at Binghamton Computer Science Department.

This article has been published as part of *BMC Genomics *Volume 14 Supplement 7, 2013: Selected articles from the Second IEEE International Conference on Computational Advances in Bio and Medical Sciences (ICCABS 2012): Genomics. The full contents of the supplement are available online at http://www.biomedcentral.com/bmcgenomics/supplements/14/S7
